# Cardiorespiratory fitness is strongly linked to metabolic syndrome among physical fitness components: a retrospective cross-sectional study

**DOI:** 10.1186/s40101-020-00241-x

**Published:** 2020-10-01

**Authors:** Bokun Kim, Minjae Ku, Tanaka Kiyoji, Tomonori Isobe, Takeji Sakae, Sechang Oh

**Affiliations:** 1 Faculty of Medicine, University of Tsukuba, 1-1-1 Tennodai, Tsukuba, Ibaraki, 305-8575 Japan; 2 Department of Sports Health Care, Inje University, Gimhae, Republic of Korea; 3 Department of Leisure Sports, Masan University, Masan, Republic of Korea; 4 Faculty of Health and Sports Science, University of Tsukuba, Tsukuba, Ibaraki Japan

**Keywords:** Cardiorespiratory fitness, Metabolic syndrome, Physical fitness

## Abstract

**Background:**

Maintaining a good level of physical fitness from engaging in regular exercise is important for the treatment and prevention of metabolic syndrome (MetS). However, which components constitutive of physical fitness confer the greatest influence remains controversial. This retrospective cross-sectional study aimed to investigate the association between MetS and physical fitness components including cardiorespiratory fitness, muscle strength, flexibility, and agility and to identify which physical fitness components have the largest influence on MetS.

**Methods:**

A total of 168 Japanese adult males aged 25–64 years were allocated into non-MetS, pre-MetS, and MetS groups according to the criteria recommended by the Japanese Society of Internal Medicine. Anthropometric measurement of body composition by whole-body dual-energy X-ray absorptiometry and measures related to MetS, including waist circumference, triglyceride level, high-density lipoprotein cholesterol level, blood pressure, glucose level, and physical fitness components, were assessed. For evaluation of cardiorespiratory fitness, muscle strength, flexibility, agility, and balance, maximal oxygen consumption (VO_2peak_) and oxygen uptake at anaerobic threshold (VO_2AnT_), handgrip strength and vertical jumping, trunk extension and flexion, stepping side to side, and single-leg balance task with the eyes closed were assessed, respectively.

**Results:**

A progressive tendency of increasing body weight, body mass index, whole-body lean and fat mass, percentage of whole-body fat mass, trunk lean and fat mass, percentage of trunk fat mass, arm fat mass, waist circumference, triglyceride level, systolic and diastolic blood pressure, and blood glucose level from the non-MetS group to the MetS group was significant (*P* < 0.05). Conversely, the cardiorespiratory endurance parameters VO_2peak_ and VO_2AnT_ and the high-density lipoprotein cholesterol level showed a progressively decreasing tendency across the groups (*P* < 0.01). In addition, a VO_2peak_ below 29.84 ml·kg·min^−1^ (*P* = 0.028) and VO2_AnT_ below 15.89 ml·kg·min^−1^ (*P* = 0.011) were significant risk components for pre-MetS and MetS. However, there was no significant tendency with respect to muscle strength, agility, and flexibility.

**Conclusion:**

Cardiorespiratory fitness is strongly linked to metabolic syndrome among physical fitness components

## Introduction

The metabolic syndrome (MetS) is a constellation of chronic metabolic and cardiovascular risk components for central obesity, dyslipidemia, hypertension, and impaired glucose tolerance [1, 2]. According to the Japan Ministry of Health, Labour and Welfare, a total of 45.6% of Japanese adult men meet the criteria for pre-metabolic syndrome (pre-MetS) or MetS [3]. Moreover, it is expected that the prevalence of MetS will continue to increase in Japan because of a dietary propensity toward energy-dense nutrient-poor foods more common in Western countries and a chronic insufficiency of exercise. Considering these situations, establishing a countermeasure that is applicable in clinical practice is very important.

As a countermeasure to MetS, change to a more healthy lifestyle, including modifications in diet and physical activity, are recommended [2, 4]. Several previous studies have demonstrated that maintaining a good level of physical fitness by engaging in regular exercise is important for the treatment and prevention of MetS [4–6]. However, although it is widely accepted that enhanced physical fitness is beneficial for MetS, which components constitutive of physical fitness that exert the greatest influence on MetS, including cardiorespiratory fitness, muscle strength, agility, and flexibility, remains controversial. Mason et al. (2007) reported the need for an integrative evaluation of physical fitness, including cardiorespiratory fitness, muscle strength, and flexibility [7]. Jurca et al. (2004) reported that both cardiorespiratory fitness and muscle strength are inversely related to MetS and especially emphasized that muscular strength has a more protective influence against MetS than cardiorespiratory fitness [8]. By contrast, a more recent study by Misigoj-Durakovic et al. (2016) reported that cardiorespiratory fitness exerted a strong protective influence against MetS but that the influence of muscle strength was less obvious [9]. This discrepancy between previous reports will likely lead to misunderstandings regarding the influence of physical fitness and inappropriate exercise prescriptions for MetS. Accordingly, the association between MetS and physical fitness should be further investigated and clarified.

Therefore, we conducted a cross-sectional study to investigate the association between MetS and physical fitness. In other words, we aimed to identify which physical fitness factor among cardiorespiratory fitness, muscle strength, flexibility, and agility has the largest influence on MetS. To this end, we evaluated differences in physical fitness among non-Mets, pre-MetS, and MetS groups of individuals.

## Materials and methods

### Subjects

**Fig. 1:**
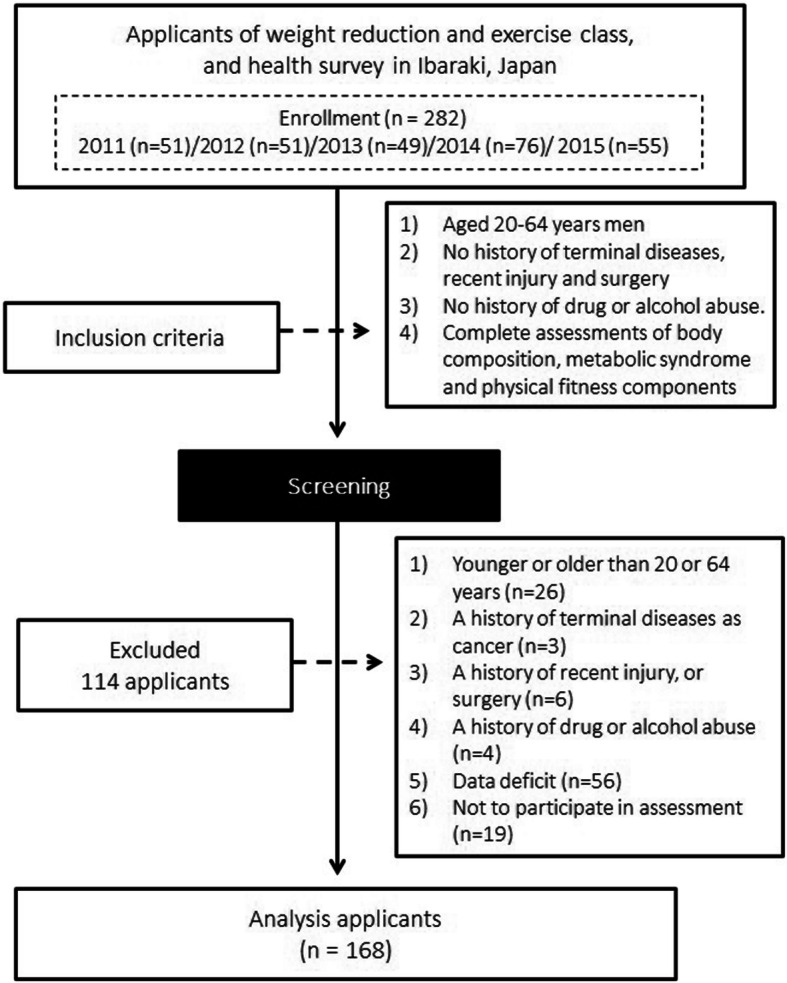
Flow diagram of study participants’ enrollment and classification

A total of 282 Japanese adult men were recruited via an advertisement placed in local newspapers in Ibaraki prefecture from 2011 through 2015. Every applicant had to meet the following criteria to be included in the study: (a) an adult male aged 20–64 years, (b) no terminal disease or recent injury or surgery, and (c) no history of drug or alcohol abuse. As shown Fig. 1, we excluded applicants who were not eligible for the present study (*n* = 114), were older than 64 years or younger than 20 years (*n* = 26), had a history of a terminal disease such as cancer (*n* = 3), had a history of a recent injury or surgery (*n* = 6), had a history of drug or alcohol abuse (*n* = 4), had incomplete data (*n* = 56), or did not participate in the assessment (*n* = 19). Of the initial 282 applicants, 168 were finally included in the analysis, all of whom read and signed the informed written consent form that was approved by the Institutional Review Board.

This study was carried out in accordance with the guidelines proposed in the Declaration of Helsinki, and the study protocol was reviewed and approved by the Ethics Committee of the University of Tsukuba in Japan.

### Anthropometry and body composition

Height was measured to the nearest 0.1 cm using a wall-mounted stadiometer (YG-200; Yagami, Nagoya, Japan), and body weight was measured to the nearest 0.1 kg using a digital scale while the subject wore light clothing and no shoes (TBF-551; Tanita, Tokyo, Japan). Body mass index (BMI) was calculated as the weight (kg) divided by the square of the height (m). Body composition was assessed using whole-body dual-energy X-ray absorptiometry (QDR 4500; Hologic, Bedford, MA, USA). Hologic software was used to estimate the fat and lean masses (kg) and the percentage of fat mass. Extended analyses were performed to obtain separate fat and lean masses and percentages of fat mass for the arms, legs, and trunk [10, 11].

### Symptoms of metabolic syndrome

The criteria recommended by the Japanese Society of Internal Medicine were used to diagnose pre-MetS and MetS [12]. Abdominal obesity, which is defined by a waist circumference of 85 cm or larger, is a criterion that must be met for these diagnoses. Additional criteria include the following: (a) dyslipidemia: the triglyceride level is higher than 150 mg·dL^−1^ or the high-density lipoprotein cholesterol (HDLC) level is lower than 40 mg·dL^−1^; (b) hypertension: the systolic blood pressure (SBP) is higher than 130 mmHg or the diastolic blood pressure (DBP) is higher than 85 mmHg; and (c) hyperglycemia: the fasting blood glucose (FPG) level is higher than 110 mg·dL^−1^. Based on these criteria, the 168 subjects who had none, one, and two or more of these conditions were allocated to the non-MetS (55 subjects), pre-MetS (57 subjects), and MetS (56 subjects) groups, respectively. For the waist circumference measurement, the subjects were asked to stand upright without outerwear while the horizontal circumference around the umbilicus was measured using a tape measure. Blood samples were collected in the morning after the participants fasted for 8 h or more. The triglyceride (TG), HDLC, and FPG levels were analyzed by enzymatic colorimetric, heparin-manganese precipitation, and glucose oxidase methods, respectively. Well-experienced observers assessed the SBP and DBP using a standard mercury sphygmomanometer (cuff size 14 cm × 47 cm) that was placed on the right arm of the seated subjects after they rested for at least 10 min.

### Physical fitness components

To evaluate physical fitness components including cardiorespiratory fitness, muscle strength, agility, flexibility, and balance, the following measurements were conducted. VO_2peak_ and VO_2AnT_ were assessed to evaluate cardiorespiratory fitness. A graded exercise test using a cycle ergometer (818E; Monark, Stockholm, Sweden) was carried out to assess VO_2peak_ and VO_2AnT_; a multistage incremental load protocol in which the friction load was increased by 0.25 kP min^−1^ was used. The pedal revolution remained constant at 60 rpm. A metabolic assessment unit (Oxycon Alpha System; Mijnhardt, Breda, the Netherlands) was used to analyze the expired gas parameters. VO_2AnT_ was detected by the rising point of VCO_2_ to VO_2_ (V slope method). Handgrip strength for both hands was assessed as a measure of upper-limb muscle strength. Subjects gripped a dynamometer (Grip-D, T.K.K. 5401; Takei Scientific Instruments, Tokyo, Japan) in each hand, alternately, with maximum effort, while they lowered their arm naturally to the side of their body. Vertical jumping was also performed for the assessment of lower-limb muscle strength. The subjects placed their feet on a circular board attached to a dynamometer (Jump-MD, T.K.K. 5106; Takei Scientific Instruments) and attached the dynamometer around their waist. Subjects leaped vertically as high as possible using a knee countermovement, landing on the circular board attached to the dynamometer. For the agility measurement, subjects performed a side-to-side stepping movement. The subjects stood in the middle of three lines (separated by an interval of 1 m) and moved to the left or right side after a signal was given. After the subject’s foot touched a line, they moved back to the middle line and then moved to the other side. This movement indicated the completion of one repetition, and the subjects tried to achieve as many repetitions as possible in 20 s. Flexibility was measured using a standing trunk flexion meter (Flexion-A, T.K.K. 1229; Takei Scientific Instruments), and trunk extension was measured using a trunk extension meter (Extension-A, T.K.K. 1229; Takei Scientific Instruments). For the evaluation of balance, the single-leg balance task with the eyes closed was performed. Subjects were asked to put their hands at their waist and then raise their preferred foot approximately 20 cm above the floor for 60 s. The time was assessed from the hold position until (a) the raised foot touched either the supporting leg or the ground, (b) the supporting leg shifted, or (c) either one or both hands moved away from the waist.

### Statistical analysis

Measurements were expressed as means ± standard deviation. One-way analysis of variance (ANOVA) was used to analyze the differences in the anthropometric, body composition, MetS, and physical fitness measures across the three groups. The Bonferroni post hoc test was used when the ANOVA results showed significant differences (*P* < 0.05). The Jonckheere-Terpstra test was used to assess the trends among the values in the three groups. The standardized statistic (SS) in the test shows the quantitative change of the dependent parameter according to the change of the independent parameter. The trend test was two-tailed, with a significance level of *P* < 0.05. Logistic regression was employed to determine the associations among VO2_peak_, VO_2AnT_, and pre-MetS and MetS. The results were presented as odds ratios with 95% confidence intervals. SPSS software, version 25.0 (IBM, Armonk, NY, USA), was used for the statistical analyses.

## Results

Table 1 shows the anthropometric and body composition measurements and trends among the three groups (non-MetS, pre-MetS, and MetS). The ANOVA results demonstrated that the height, weight, BMI, whole-body lean mass, and fat mass values in the non-MetS group were noticeably lower than those in the pre-MetS and MetS groups, although significant differences were not detected between the pre-MetS and MetS groups. There was no significant difference in age between the groups, and the percentage of whole-body fat mass in the pre-MetS group was noticeably higher than that in the non-MetS group. The trend test detected a progressive tendency of the height (SS = 3.56, *P* < 0.01), weight (SS = 5.86, *P* < 0.01), BMI (SS = 4.99, *P* < 0.01), whole-body lean mass (SS = 3.01, *P* < 0.01) and fat mass (SS = 3.20, *P* < 0.01), and the percentage of whole-body fat mass (SS = 2.09, *P* < 0.05) to increase from the non-MetS group to the MetS group. For age, no tendency among the groups was detected. The multiple comparison results demonstrated that the values of trunk lean and fat mass in the non-MetS group were noticeably lower than those in the pre-MetS and MetS groups, but a significant difference was not detected between the pre-MetS and MetS groups. There was no significant difference in the percentage of trunk fat mass between the groups. A progressive trend of increasing trunk lean mass (SS = 3.69, *P* < 0.01), trunk fat mass (SS = 3.47, *P* < 0.01), and percentage of trunk fat mass (SS = 2.27, *P* < 0.05) from the non-MetS group to the MetS group was detected. The post hoc test demonstrated that the arm and leg lean masses, and arm fat mass in the pre-MetS group were noticeably higher than those in the non-MetS group, and the leg fat mass in the pre-MetS group were noticeably higher than those in the non-MetS and MetS groups. However, a significant difference was not detected in the percentage of leg and arm fat masses between the groups. The trend test indicated no significant tendency in any of the arm and leg parameters, except for arm fat mass (SS = 2.06, *P* < 0.05).

**Table 1:** Anthropometric and body composition characteristics and trends among three groups

	Non-MetS group (A) (95% CI)	Pre-MetS group (B) (95% CI)	MetS group (C) (95% CI)	Post hoc	SS^b^	*P* for trend^b^
	(*n* = 55)	(*n* = 57)	(*n* = 56)			
Age, year	50.5 ± 9.7 (47.9, 53.1)	49.4 ± 9.6 (46.8, 51.9)	49.7 ± 7.9 (47.6, 52.8)	NS	− 0.65	= 0.51
Height, cm	169.1 ± 5.5 (167.6, 170.6)	172.2 ± 6.5 (170.4, 173.9)	172.8 ± 5.4 (171.3, 174.2)	A < B, C	3.56	< 0.01
Weight, kg	73.8 ± 10.4 (71.0, 76.6)	86.2 ± 13.9 (82.5, 89.8)	86.8 ± 9.7 (84.3, 89.4)	A < B, C	5.86	< 0.01
BMI, kg/m^2^	25.8 ± 3.3 (24.9, 26.7)	28.9 ± 3.5 (28.0, 29.9)	29.1 ± 3.2 (28.3, 30.0)	A < B, C	4.99	< 0.01
WLM, kg	59.6 ± 8.1 (57.4, 61.8)	63.8 ± 7.2 (61.9, 65.7)	63.9 ± 7.6 (61.9, 65.9)	A < B, C	3.01	< 0.01
WFM, kg	19.1 ± 5.3 (17.6, 20.5)	22.6 ± 5.6 (21.1, 24.1)	22.3 ± 5.3 (20.9, 23.8)	A < B, C	3.20	< 0.01
%WFM	23.7 ± 4.1 (22.6, 24.8)	25.6 ± 4.0 (24.5, 26.7)	25.3 ± 3.7 (24.3, 26.3)	A < B	2.09	< 0.05
TLM, kg	29.1 ± 3.9 (28.0, 30.1)	31.8 ± 3.9 (30.7, 32.8)	32.1 ± 4.0 (31.0, 33.2)	A < B, C	3.93	< 0.01
TFM, kg	9.9 ± 3.1 (9.0, 10.7)	12.1 ± 3.9 (11.1, 13.1)	12.2 ± 3.3 (11.3, 13.1)	A < B, C	3.65	< 0.01
%TFM	24.8 ± 4.8 (23.5, 26.1)	26.9 ± 5.5 (25.5, 28.4)	27.0 ± 4.7 (25.8, 28.3)	NS	2.33	< 0.05
ALM, kg	6.0 ± 1.9 (5.5, 6.5)	6.9 ± 1.1 (6.6, 7.1)	6.3 ± 1.6 (5.9, 6.8)	A < B	1.23	= 0.22
AFM, kg	1.9 ± 0.7 (1.7, 2.1)	2.5 ± 0.7 (2.3, 2.6)	2.2 ± 0.7 (2.0, 2.4)	A < B	2.05	< 0.05
%AFM	24.0 ± 4.9 (22.7, 25.4)	25.8 ± 4.8 (24.5, 27.0)	25.2 ± 4.0 (24.1, 26.3)	NS	1.34	= 0.18
LLM, kg	17.9 ± 5.3 (16.5, 19.3)	20.4 ± 3.1 (19.5, 21.2)	18.5 ± 4.6 (17.3, 19.8)	A < B	0.98	= 0.35
LFM, kg	5.3 ± 2.0 (4.8, 5.9)	6.7 ± 1.8 (6.3, 7.2)	5.8 ± 2.1 (5.3, 6.4)	A < B, B > C	1.39	= 0.17
%LFM	22.6 ± 4.5 (21.4, 23.8)	24.3 ± 4.1 (23.2, 25.3)	23.2 ± 4.0 (22.1, 24.3)	NS	0.78	= 0.43

NOTES: Values are means±SD

*95% CI* 95% confidence interval, *NS* not significant, *non-MetS* non-metabolic syndrome, *pre-MetS* pre-metabolic syndrome, *MetS* metabolic syndrome, *SS* standardized statistic, *BMI* body mass index, *WLM* whole body lean mass, *WFM* whole body fat mass, *%WFM* percentage of whole body fat mass, *TLM* trunk lean mass, *TFM* trunk fat mass, *%Trunk mass* percentage of trunk fat mass, *ALM* arm lean mass, *AFM* arm fat mass, *%AFM* percentage of arm fat mass, *LLM* leg lean mass, *LFM* leg fat mass, *%Leg fat mass* percentage of leg fat mass

^b^Jonckheere-Terpstra test was used to assess the trend among three groups

Table 2 presents the characteristics of the symptoms of MetS and trends among the three groups. The multiple comparison results indicated significant differences in all symptoms of MetS across the three groups. The waist circumference values in pre-MetS and MetS groups were higher than those in the non-MetS group but did not differ between pre-MetS and MetS groups. The TG levels in the non-MetS and pre-MetS groups were lower than those in the MetS group but did not differ between non-MetS and pre-MetS groups. Regarding the levels of SBP and DBP, the groups were ranked in the ascending order non-MetS, pre-MetS, and MetS. The HDLC levels were not different between pre-MetS and MetS groups but were lower in pre-MetS and MetS groups than in the non-MetS group. The blood glucose levels were different between non-MetS and pre-MetS groups but were noticeably lower in the non-MetS and pre-MetS groups than in the MetS group. According to the trend analysis, the increase in waist circumference, TG level, SBP, DBP, and FPG level from the non-MetS group to the MetS group was significant (SS = 4.70, 7.79, 7.54, 8.61, and 5.98, respectively; *P* < 0.01 for all), but the HDLC level trended in the opposite direction from the other parameters (SS = − 4.26, *P* < 0.01).

**Table 2:** Characteristics of components of metabolic syndrome and trends among three groups

	Non-MetS (A) (95% CI)	Pre-MetS (B) (95% CI)	MetS (C) (95% CI)	Post hoc	SS^b^	*P* for trend^b^
	(*n* = 55)	(*n* = 57)	(*n* = 56)			
Abdominal obesity						
WC, cm	93.0 ± 8.4 (90.7,95.3)	101.1 ± 8.9 (98.8,103.5)	101.5 ± 7.0 (99.7,103.4)	A < B, C	5.04	< 0.01
%Abdominal obesity	80.0	100.0	100.0			
Dyslipidemia						
TG, mg·dL^-1^	90.8 ± 38.7 (80.4,101.3)	137.5 ± 96.9 (111.7,163.2)	247.5 ± 166.6 (202.9,292.1)	A, B < C	8.17	< 0.01
HDLC, mg·dL^-1^	56.3 ± 9.2 (53.8,58.8)	48.8 ± 10.9 (45.9,51.8)	47.3 ± 10.7 (44.5,50.2)	A > B, C	-4.44	< 0.01
%Dyslipidemia	1.8	31.6	85.7			
Hypertension						
SBP, mmHg	116.8 ± 9.2 (114.3,119.3)	127.0 ± 13.6 (123.4,130.6)	135.6 ± 12.1 (132.4,138.9)	A < B < C	7.34	< 0.01
DBP, mmHg	77.1 ± 6.9 (75.2,78.9)	86.6 ± 9.2 (84.1,89.0)	93.5 ± 8.3 (91.2,95.6)	A < B < C	8.68	< 0.01
%Hypertension	9.1	63.2	98.2			
Hyperglycemia						
FPG, mg/dL	90.7 ± 6.7 (88.9,92.5)	94.5 ± 9.0 (92.1,96.9)	114.1 ± 37.5 (104.1,124.2)	A, B < C	6.13	< 0.01
%Hyperglycemia	0.0	3.5	42.6			

NOTES: Values are means±SD

*95% CI* 95% confidence interval, *non-MetS* non-metabolic syndrome, *pre-MetS* pre-metabolic syndrome, *MetS* metabolic syndrome, *SS* standardized statistic, *WC* waist circumstance, *%Abdominal obesity* percentage of abdominal obesity, *TG* triglycerides, *HDLC* high-density lipoprotein cholesterol, *%Hypertension* percentage of hypertension, *FPG* fasting plasma glucose, *%Hyperglycemia* percentage of hyperglycemia

^b^Jonckheere-Terpstra test was used to assess the trend among three groups

Table 3 shows the results for the components of physical fitness and trends among the three groups. ANOVA results demonstrated that there were no significant differences between groups for all components of physical fitness, except for VO_2peak_ and VO_2AnT_. The values of VO_2peak_ and VO_2AnT_ in pre-MetS and MetS groups were not different and were noticeably lower than the values in the non-MetS group. The trend test revealed that there were no tendencies in any of the components of physical fitness between the groups, except in VO_2peak_ and VO_2AnT_. These two parameters showed a progressively decreasing tendency from the non-MetS group to the MetS group (SS = −3.68 and − 3.78, respectively; *P* < 0.01 for both).

**Table 3:** Characteristics of components of physical fitness and trends among three groups

	Non-MetS (A) (95% CI)	Pre-MetS (B) (95% CI)	MetS (C) (95% CI)	Post hoc	SS^b^	*P* for trend^b^
	(*n* = 55)	(*n* = 57)	(*n* = 56)			
VO_2AnT_ (ml·kg·min^-1^)	19.12 ± 4.16 (17.99, 20.24)	16.51 ± 3.27 (15.64, 17.38)	16.54 ± 5.01 (15.20, 17.88)	A > B, C	− 3.68	< 0.01
VO_2_max (ml·kg·min^-1^)	33.70 ± 5.49 (32.22, 35.19)	30.51 ± 5.02 (29.18, 31.84)	29.60 ± 5.33 (28.17, 31.03)	A > B, C	− 3.78	< 0.01
Right hand grip strength (kg)	43.43 ± 7.15 (41.49, 45.36)	44.12 ± 8.09 (41.98, 46.27)	43.96 ± 5.58 (42.47, 45.45)	NS	0.45	= 0.66
Left hand grip strength (kg)	41.76 ± 6.63 (39.96, 43.55)	43.83 ± 7.66 (41.80, 45.86)	41.43 ± 6.22 (39.76, 43.09)	NS	0.09	= 0.93
Stepping side-to-side (n/20 s)	41.04 ± 7.92 (38.90, 43.18)	38.41 ± 5.82 (36.79, 39.88)	38.43 ± 5.92 (36.84, 40.01)	NS	− 1.64	= 0.10
Trunk flexion (cm)	0.21 ± 9.76 (-2.43, 2.85)	-3.09 ± 9.58 (-5.63, -0.54)	− 2.76 ± 9.39 (− 5.27, − 0.24)	NS	− 1.55	= 0.12
Trunk extension (cm)	40.49 ± 9.45 (37.94, 43.04)	38.56 ± 8.47 (36.32, 40.81)	41.77 ± 8.83 (39.40, 44.13)	NS	0.61	= 0.54
Vertical jump (cm)	42.80 ± 7.91 (40.66, 44.94)	41.95 ± 6.95 (40.10, 43.79)	42.73 ± 8.12 (40.56, 44.91)	NS	− 0.13	= 0.89
Single-leg balance with eyes closed (s)	19.13 ± 19.30 (13.91, 24.34)	12.24 ± 12.86 (8.83, 15.65)	12.96 ± 13.87 (9.25, 16.68)	NS	− 1.75	= 0.08

NOTES: Values are means±SD

*95% CI* 95% confidence interval, *NS* not significant, *non-MetS* non-metabolic syndrome, *pre-MetS* pre-metabolic syndrome, *MetS* metabolic syndrome, *SS* standardized statistic

^b^Jonckheere-Terpstra test was used to assess the trend among three groups

**Fig. 2:**
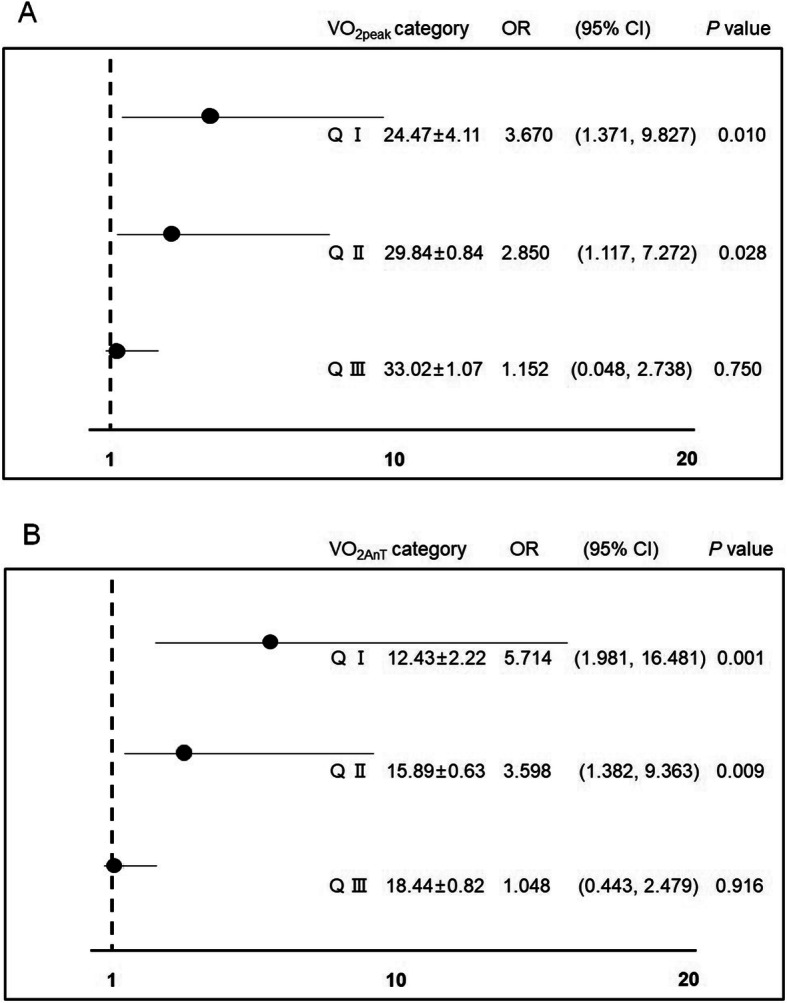
Association between cardiorespiratory fitness and pre-MetS and MetS. **a** Association between VO_2peak_ and pre-MetS and MetS. **b** Association between VO_2AnT_ and pre-MetS and MetS. pre-MetS pre-metabolic syndrome, MetS metabolic syndrome, VO_2peak_ peak oxygen consumption, VO_2AnT_ oxygen uptake at anaerobic threshold, Q I quartile I, Q II quartile II, Q III quartile III

The associations between MetS and VO_2peak_ and VO_2AnT_ are shown in Fig. 2a, b, respectively. When the results were divided into quartile depending on the VO_2peak_ values, group quartile II (Q II), whose VO_2peak_ was 29.84 ml kg·min^−1^, had 2.85-fold increased odds and group quartile I (Q I), whose VO_2peak_ was 24.47 ml·kg·min^−1^, had 3.67-fold increased odds for pre-MetS and MetS compared with group quartile IV (Q IV), whose VO_2peak_ was 37.86 ml kg·min^−1^ (Fig. 2a). Similarly, when the results were categorized into quartile based on the VO_2AnT_ values, group Q II, whose VO_2AnT_ was 15.89 ml kg·min^−1^, had 3.43-fold increased odds and group Q I, whose VO_2AT_ was 12.43 ml kg·min^−1^, had 5.46-fold increased odds for pre-MetS and MetS compared with group Q IV, whose VO_2AnT_ was 22.82 ml·kg·min^−1^ (Fig. 2b).

## Discussion

The purpose of this retrospective cross-sectional study was to investigate the association between MetS and the physical fitness component cardiorespiratory fitness, muscle strength, flexibility, and agility. The primary findings of this study are that the two cardiorespiratory fitness parameters, VO_2peak_ and VO_2AnT,_ showed a progressively decreasing tendency from the non-MetS group to the MetS group. A VO_2peak_ value below 29.84 ml kg min^−1^ and VO_2AnT_ value below 15.89 ml·kg·min^−1^ were significant risk components for pre-MetS and MetS. There was no significant tendency with respect to muscle strength, agility, or flexibility. These findings suggest that cardiorespiratory fitness is strongly linked to MetS among physical fitness components.

Previous studies have reported that as body weight increases, both lean and fat mass increase and that an increase in fat mass, especially abdominal fat mass and percentage of whole-body fat mass is related to several health conditions regardless of several components such as the person’s age, sex, race, or BMI [13–15]. In this study, as MetS developed, the weight, whole-body lean mass, and fat mass simultaneously increased, and parameters such as trunk fat mass, percentage of trunk fat mass, and waist circumference, which indicate abdominal obesity increased. These findings may indicate that abdominal obesity is strongly related to the prevalence of dyslipidemia, hypertension, and hyperglycemia, as reported in previous studies. However, even though 80.0% of the subjects in the non-MetS group were considered to show abdominal obesity, the incidence rates of dyslipidemia (1.8%), hypertension (9.1%), and hyperglycemia (0.0%) were relatively low compared with those in the pre-MetS and MetS groups. These findings suggest that there may be a factor that prevents and reduces the prevalence of pre-MetS and MetS.

There were no significant tendencies in the ten components of physical fitness, except for VO_2peak_ and VO_2AnT_. These two parameters of cardiorespiratory fitness exhibited a significant decreasing tendency from the non-MetS to pre-MetS and MetS groups, and the values (33.70 ml kg·min^−1^ in VO_2peak_ and 19.12 ml kg·min^−1^ in VO_2AnT_) in the non-MetS group were noticeably higher than those in the pre-MetS and MetS groups. Additionally, the lowest two quantiles (average 29.84 and 24.47 ml kg·min^−1^) in VO_2peak_ had 2.85- and 3.67-fold increased odds, and similarly the lowest two quantiles (average 15.89 and 12.43 ml·kg·min^−1^) in VO_2AnT_ had 3.43- and 5.46-fold increased odds compared with the highest quantile. The Japanese recommendations suggest maintaining a VO_2peak_ higher than 33 ml kg·min^−1^. This value is the average of the lowest values that noticeably increase the incidence of lifestyle-related diseases, such as MetS. In the present study, the non-MetS group was the only group that met this recommendation. Previous studies have reported the possible mechanisms by which high cardiorespiratory fitness prevents and reduces the prevalence of MetS; low cardiorespiratory fitness may lead to decreased arterial compliance and worse insulin resistance and may cause hyperglycemia and reduced physical activity [16–18]. Based on the recommendations, results of previous studies, and the findings of this study, it seems that high cardiorespiratory fitness prevents and decreases the prevalence of MetS.

For many years, muscle strength has been widely accepted as an independent predictor of MetS [19]. However, the findings of this study were not consistent with this perception. There were no differences among the groups, and no tendencies were detected with respect to muscle strength. Additionally, more recent studies including those by Misigoj-Durakovic et al. (2016) and Kim et al. (2011) also reported results that contradict this outdated viewpoint [9, 20]. According to those reports, after cardiorespiratory fitness was accounted for, the inverse relationship between MetS and muscle strength was not present or was attenuated [9, 20]. The results from the current study and recent studies indicate that the influence of muscle strength on the prevalence of MetS is not large and that muscle strength is not strongly linked to MetS.

With respect to agility and flexibility, significant tendencies among the groups were not detected in this study. Kim et al. (2015) reported that excessive fat mass increases in men with obesity are a physical burden on the musculoskeletal system; as obesity develops, the whole-body fat mass increases more than does the whole-body lean mass [13]. Since the increased physical burden causes people to carry an extra load during physical activity, their agility is reduced. Similarly, a negative association between flexibility and waist circumference, resulting from excessive fat increases acting as a physical obstacle when the trunk is flexed or extended, has been reported [21]. Consequently, it is accepted that excessive fat increases have negative influences on agility and flexibility because they act as a physical burden. However, it is difficult to diagnose MetS on the basis of agility and flexibility. Decreased agility and flexibility are consequences of an increased physical burden but not internal changes such as arterial compliance and insulin resistance.

Concerning clinical application, first, the use of cutoff values of 29.84 ml·kg·min^−1^ in VO_2peak_ and 15.89 ml·kg·min^−1^ in VO_2AnT_ can be utilized to screen high-risk individuals for MetS. Early detection of such individuals may contribute to reducing the prevalence of cardiovascular disease and diabetes. Second, to motivate regular exercise in individuals and prevent injuries caused by excessive exercise, exercise prescriptions including the mode, frequency, and quantity should be carefully considered. The values can be employed as a standard to prevent side effects derived from excessive exercise and to evaluate whether the exercise prescription is appropriate for the prevention and improvement of MetS.

This retrospective cross-sectional study has two limitations. First, the design is cross-sectional, which limits the potential to make causal inferences from the observed associations. Second, the applicability of the findings is limited because only adult men were selected for the study. Consequently, a long-term follow-up study involving various subjects is needed to confirm the current findings.

## Conclusions

The two cardiorespiratory fitness parameters, VO_2peak_ and VO_2AnT_, showed a progressively decreasing tendency from the non-MetS group to the MetS group. A VO_2peak_ value below 29.84 ml kg min^−1^ and VO_2AnT_ value below 15.89 ml kg min^−1^ were significant risk components for pre-MetS and MetS. However, significant tendencies in muscle strength, agility, and flexibility among the groups were not detected. These findings suggest that cardiorespiratory fitness is strongly linked to MetS among physical fitness components.
